# Management of validation of HPLC method for determination of acetylsalicylic acid impurities in a new pharmaceutical product

**DOI:** 10.1038/s41598-021-99269-x

**Published:** 2022-01-06

**Authors:** Małgorzata Kowalska, Magdalena Woźniak, Michał Kijek, Paulina Mitrosz, Jerzy Szakiel, Paweł Turek

**Affiliations:** 1 Department of Management and Product Quality, Faculty of Chemical Engineering and Commodity Science, Kazimierz Pulaski University of Technology and Humanities, 27 Chrobrego St, 26-600 Radom, Poland; 2 Medicofarma S.A., 13 Tarnobrzeska St, 26-613 Radom, Poland; 3 Department of Non-Food Product Quality and Safety, Cracow University of Economics, Rakowicka St. 27, 31-510 Cracow, Poland

**Keywords:** Bioanalytical chemistry, Chemistry publishing

## Abstract

The work mainly focused on a validation of the method for determining the content of salicylic acid and individual unknown impurities in new pharmaceutical product—tablets containing: 75, 100 or 150 mg of acetylsalicylic acid and glycine in the amount of 40 mg for each dosage. The separation of the components was carried out by means of HPLC, using a Waters Symmetry C18 column (4.6 × 250 mm, 5 μm) as the stationary phase. The mobile phase consisted of a mixture of 85% orthophosphoric acid, acetonitrile and purified water (2:400:600 V/V/V). Detection was carried out at a wavelength of 237 nm, with a constant flow rate of 1.0 ml min^−1^. In order to verify the method, linearity, precision (repeatability and reproducibility), accuracy, specificity, range, robustness, system precision, stability of the test and standard solution, limit of quantification and forced degradation were determined. Validation tests were performed in accordance with ICH (International Conference on Harmonisation of Technical Requirements for Registration of Pharmaceuticals for Human Use) guidelines. The method was validated successfully. It was confirmed that the method in a tested range of 0.005–0.40% salicylic acid with respect to acetylsalicylic acid content is linear, precise and accurate.

## Introduction

Practical aspects resulting from the implementation of the quality management system in companies in various fields (e.g., information flow, management of measurement equipment, standard operating procedures or dealing with deviations) allow to obtain a high-quality product. A fundamental role in the proper approach to the requirements imposed on companies in the pharmaceutical industry by the market is their compliance with ISO standards relating to quality management.

The basis of quality control, not only in the pharmaceutical industry but also in the food and cosmetics industry, is properly developed and characterized methods and testing tools. Accurate verification of analytical methods is necessary to ensure high quality of products, which primarily affects the safety of their use. For this purpose, validation is performed, which is the confirmation of meeting the requirements for a specific use or application of a method, by providing objective evidence. The validation process in the pharmaceutical industry is mandatory by law. The concept has also been popularised by quality management systems, mainly ISO 9000 standards, and refers to the validation of analytical methods as well as processes and control measures. Therefore, it is reasonable and important to conduct validation, which unfortunately is often treated as a complicated and labour-intensive procedure.

The primary direction of the development of analytical procedures of impurities control in pharmaceutical products is the aspiration of determination of the lowest concentrations of substances in the tested samples, which are within the tolerance limits^1^. Each pharmaceutical product launched into the market should be of the highest quality and, above all, safety of use. Agreement with the above-mentioned priorities is an important issue for technologists, manufacturers and potential patients^2^. The safety of a medicinal product depends not only on the toxicological properties of active substances, but also on the profile of impurities present, e.g., in raw materials. Usually, impurity of an active substance in a medicinal product is a compound which has no chemical entity defined as a drug substance. Impurities may be formed as intermediates of the synthesis reaction of an active substance or they may be the degradation products. Therefore, often the content of impurities in a pharmaceutical product refers to its quality, which may impose a risk to patient safety^3^.

Pharmaceutical industry manufacturers are required to validate analytical methods in order to ensure the reliability of the results obtained. Detailed information is specified in the guidelines of the International Conference on Harmonisation of Technical Requirements for Registration of Pharmaceuticals for Human Use (ICH Q2A-B) in a form of 8 validation parameters^4^. Method validation should be completed at the early stage of the product development^5^. Innovative approach to the quality of medicinal formulations is on understanding that the quality of the product does not arise during the analytical testing, but starts at the first stage of production and lasts until the moment of packing and then proper transportation as well as storage^6^.

In the pharmaceutical industry, one of the most important elements are the guidelines for Good Manufacturing Practice (GMP), which are a part of the Total Quality Management System (TQM). These regulations include documenting of evidence that the validation was carried out within the set ranges of parameters and proceeded properly, which makes it possible to obtain pharmaceutical products that would meet the assumed requirements.

**Figure 1:**
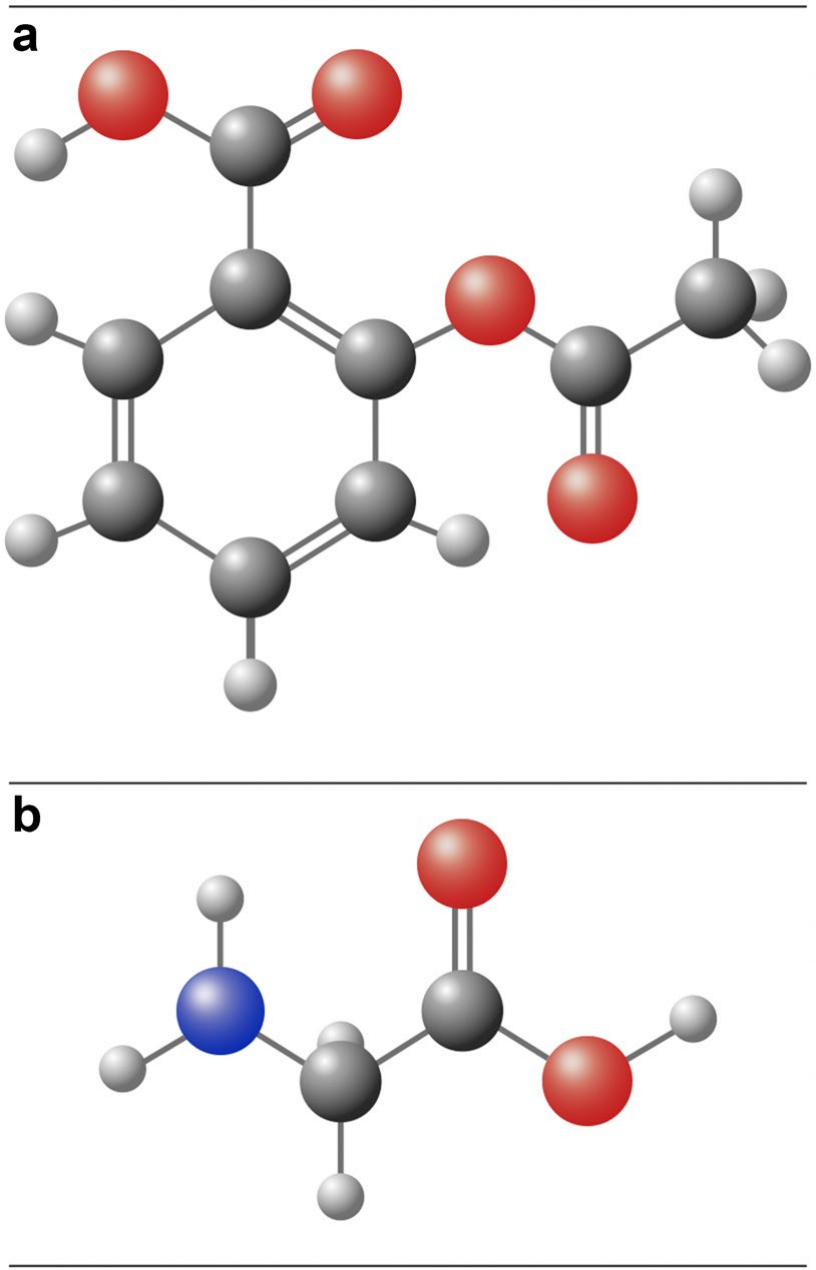
Molecule models of: (**a**) acetylsalicylic acid, (**b**) glycine.

In the presented study, the proposed medicinal product contained two active substances: acetylsalicylic acid and glycine. The structure of active substances molecules was presented in Fig. 1. Combination of these two active substances is allowed in Poland if the form of the drug is tablets that contain up to 500 mg of acetylsalicylic acid and 200 mg of glycine. Acetylsalicylic acid (ASA) represents the non-steroidal anti-inflammatory drugs and inhibits the activity of oxygenase by acetylation of the serine residue, exerting analgesic, antipyretic and anti-inflammatory effects^7,8^. It also prevents platelet aggregation, reducing the risk of ischemic stroke and heart attack^9^. Glycine (GLY) is an amino acid that acts as a neurotransmitter in the central nervous system^10^. The use of both these substances showed the improved gastrointestinal tolerability in relation to nonglycine-containing acetylsalicylic acid alternatives with respect to long-term treatment compliance^11,12^.

This study is a part of project focused on development and/or validation of the analytical methods suitable for a new pharmaceutical product. The study proposes the use of the pharmacopoeial method for the determination of the content of salicylic acid and individual unknown impurities in tablets containing: 75, 100 or 150 mg of acetylsalicylic acid and glycine in the amount of 40 mg for each dosage. The calculation of the content of individual unknown impurities was based on salicylic acid peak. The work mainly focused on a validation of the method performed in accordance with ICH guidelines.

## Materials and methods

### Materials

In the study following materials were used: pharmaceutical secondary standards (Certified Reference Material) of acetylsalicylic acid, salicylic acid and glycine (Fluka). Talc (Imifabi) and potato starch (Peepes) were used as excipients for dosage 100 mg ASA and 40 mg GLY, whereas microcrystalline cellulose (JRS Pharma) and maize starch (Roquette) were used as excipients for dosage 150 mg ASA and 40 mg GLY as well as 75 mg ASA and 40 mg GLY. Acetonitrile—gradient grade (Sigma-Aldrich) and orthophosphoric acid (Chempur). Deionized water was obtained by means of ELGA Purelab UHQ PS (High Wycombe, Bucks, UK). Syringe nylon filters (0.45 µm) (Agilent Technologies) were used.

### Methods

Following HPLC systems were used: Agilent HPLC 1200 (Santa Clara, CA, USA) and Shimadzu LC-40AD (Kioto, Japan). Chromatographic separation was performed on a Waters Symmetry C18 column, 5 µm, 250 mm × 4.6 mm. Chromatographic data were recorded and processed using Chem Station and LC solution software.

#### Chromatographic conditions

The chromatographic conditions for the determination of acetylsalicylic acid impurities in tablets were adapted from European Pharmacopoeia monograph no. 0309 of acetylsalicylic acid. The mobile phase consisted of a mixture of orthophosphoric acid, acetonitrile, water (2:400:600 V/V/V) and was filtered, and sonicated prior to use. Separation was achieved under isocratic conditions at a flow rate of 1.0 ml min^−1^ and the effluent was monitored at 237 nm. Stationary phase was maintained at 25 °C. 10 µl of each solution was injected. Run time was 50 min.

#### Standard solutions

##### Standard stock solution (1.0 mg ml^−1^)

50.0 mg of salicylic acid was dissolved and diluted to 50 ml with the acetonitrile.

##### Reference solution (0.01 mg ml^−1^)

1.0 ml of Standard stock solution was dissolved to 100 ml with the acetonitrile. The prepared solution was then filtered through a 25 mm syringe nylon filter with 0.45 μm pore size and transferred into a vial.

##### System suitability solution (SSS)

100.0 mg of acetylsalicylic acid was dissolved and diluted to 10 ml with the acetonitrile. 0.1 ml of this solution and 0.5 ml of standard stock solution were transferred to 50 ml flask and diluted to volume with acetonitrile. The prepared solution was filtered through a 25 mm syringe nylon filter with 0.45 μm pore size, then appropriate volume was transferred into a vial.

#### System suitability requirements

Resolution: minimum 2.0 between the acetylsalicylic acid and salicylic acid peaks on the SSS solution chromatogram.

#### Sample solution for tablets

20 tablets were weighed and finely powdered. An accurate weigh of the powder containing 100 mg of acetylsalicylic acid was transferred into 50 ml flask, diluted with acetonitrile, stirred and made up to volume with acetonitrile. The prepared solution was filtered through a 25 mm syringe nylon filter with 0.45 μm pore size, then appropriate volume was transferred into vial.

#### Specification limits

The specification limits were set on 0.3% w/w of salicylic acid and 0.05% w/w of individual unknown impurities (based on salicylic acid content).

#### Method validation

##### System suitability and system precision

System suitability was verified by making six consecutive injections of SSS solution. Mean, standard deviation (SD), % relative standard deviation (%RSD) of resolution factor between the acetylsalicylic acid and salicylic acid peaks, theoretical plates and of retention time were determined. System precision was demonstrated by analysis of peak areas of acetylsalicylic and salicylic acid of six SSS solution injections.

##### Linearity

The linearity was studied at six concentrations of salicylic acid: 0.0005, 0.0025, 0.005, 0.015, 0.030 and 0.040 mg ml^−1^. The working standards were prepared by appropriate dilution of salicylic acid standard solution (0.25 mg ml^−1^) to obtain concentrations in the required range. Peak area of each level was plotted against the respective concentration. Regression analysis was used to determine the slope, y-intercept and correlation coefficient (r^2^). The calibration curve was prepared in triplicate.

##### Accuracy

The accuracy of the method was determined by analysing 12 samples for each dosage.

Sample solutions of reconstituted tablet blends, containing excipients and active substances, were prepared according to the procedure described in “Methods” section. Samples were spiked with salicylic acid at the concentrations of 0.005%, 0.05%, 0.30% and 0.40% (with respect to acetylsalicylic acid content in a sample) before they were made up to volume. Three replicates were prepared for each concentration level. % Recovery of salicylic acid for each accuracy sample, mean, SD and %RSD were calculated.

##### Specificity

For interference study, mobile phase, reference solution, system suitability solution (SSS) and acetonitrile chromatograms were analysed. Furthermore, following solutions were injected for each dosage: tablet powder without acetylsalicylic acid prepared with the same excipients as those in the commercial formulation and glycine, reconstituted tablet powder, reconstituted tablet powder spiked with salicylic acid at the concentration of 0.05% and 0.30% (specification limit of an unknown impurity and salicylic acid, respectively). The chromatograms were recorded, the responses of the peaks, if any measured, were analysed and evaluated for peak interference.

##### Precision

Method precision was tested by preparing model solutions corresponding to sample solution of dosage 150 mg ASA and 40 mg GLY (active substances and excipients)—sample solution preparation was described in “Methods” section. Solutions were spiked with salicylic acid at the concentrations which were equivalent to 0.005%, 0.05% and 0.30% with respect to acetylsalicylic acid content in a sample. Three replicates were prepared for each concentration level. The analysis was performed in duplicate by Analyst 1 at the same day and using the same HPLC system to evaluate intra-day precision. For inter-day precision Analyst 2 performed analysis on a different day, using different HPLC system. %Found of salicylic acid, standard deviations in groups of results, %RSD as well as intra-day and inter-day variance were calculated.

##### Robustness

The robustness was determined to assess the effect of small but deliberate variation in the chromatographic conditions. In order to evaluate robustness of the method, model solutions were prepared corresponding to sample solution of dosage 150 mg ASA and 40 mg GLY (active substances and excipients). Solutions were spiked with salicylic acid at the concentrations which were equivalent to 0.005%, 0.05% and 0.30% with respect to acetylsalicylic acid content in a sample. Three replicates were prepared for each concentration level. Solutions were injected twice, using stationary phase temperature of 25 °C (according to the method) and 30 °C. Groups of the results were compared using the variance analysis (α = 0.05).

##### Standard and sample solutions stability

Stability of salicylic acid standard solution (reference solution) and sample solution of tablets spiked with salicylic acid at a concentration of 0.30% (with respect to acetylsalicylic acid content in a sample) were tested using solutions stored in autosampler at a temperature of 10 °C. Standard solution was injected six times, while sample solution was injected in triplicate. Calculations were based on the peak areas of injections at the start of analysis and after predetermined time. The mean values obtained were compared using the t and F tests.

##### Quantitation limit (QL)

Quantitation limit was tested by analysis of six consecutive injections of salicylic acid standard solution at the concentration of 0.0005 mg ml^−1^ in a presence of placebo and glycine. Mean, SD and %RSD of the peak area response were calculated.

##### Forced degradation

Acetylsalicylic acid was subjected to the following stress degradation: acidic, alkaline, oxidative, and hydrolytic conditions as well as thermal induced and photo degradation. Tablet placebo (tablet powder without acetylsalicylic acid prepared with the same excipients as those in a commercial formulation) was subjected to the same stress conditions. For this purpose, to each sample following materials were added (separately): 25 ml of 0.5 M HCl, 25 ml of 0.5 M NaOH (neutralized after cooling with 15 ml of 0.5 M HCl), 25 ml of purified water, 25 ml of 3% H_2_O_2_. Then samples were heated in a water bath for 1 h at 100 °C. Afterwards the samples were made up to volume of 50 ml with acetonitrile. The prepared solutions were filtered through a 25 mm nylon syringe filters with 0.45 μm pore size, and appropriate volumes were transferred into vials. Separate procedure concerned the preparation of samples subjected to UV and thermal degradation. For this purpose, the samples were transferred to petri dishes and kept in a photo stability chamber for 8 h or in an oven at 105 °C for 2 h. Following procedure was performed according to the description of the method—preparation of sample solution for tablets.

##### Statistics

Statistics were performed using Excel software (Microsoft Inc, Redmond, Wash).

## Results and discussion

### System suitability and system precision

System suitability is an important parameter which allows to ensure that the resolution and reproducibility of the chromatographic system are adequate for the analysis and whether the used method is valid or not^3,13^. %RSD for peak areas of six consecutive injections of SSS solution was found to be ≤ 2.0% (0.2% for SA and ASA) (Table 1). Peaks retention times showed slight variations. %RSD was found to be 0.1% for ASA as well as for SA. All the system suitability chromatograms’ theoretical plates were above 4000, which fulfilled acceptance criteria for this parameter. Resolution between SA and ASA peaks also showed fulfilment of the limits (above 2.0). The obtained results suggested that the conditions are adequate and method can be used in routine analysis.

**Table 1:** System suitability and system precision parameters.

Injection	Peak area		Retention time		Theoretical plates		Resolution
	ASA	SA	ASA	SA	ASA	SA	ASA and SA peaks
1	344,408	338,225	4.9	7.4	6031	4354	7.2
2	343,268	338,263	4.9	7.4	6063	4368	7.2
3	343,709	338,257	4.9	7.4	6068	4377	7.2
4	342,684	337,322	4.9	7.4	6109	4387	7.2
5	342,581	336,785	4.9	7.4	6108	4389	7.2
6	342,338	337,350	4.9	7.4	6117	4396	7.2
Mean	343,165	337,700	4.9	7.4	6083	4379	7.2
SD	788	633	0.0	0.0	34	16	0.0
%RSD	0.2	0.2	0.1	0.1	0.6	0.4	0.2

### Linearity

According to ICH guideline (ICH, 2996) linearity refers to the ability of obtaining test results, which are proportional to the concentration of analyte in the sample within a specified range. As a result of the performed analysis, linear correlation was obtained (r^2^ = 0.9997) between peak area and concentration of salicylic acid. Calibration curves were linear for concentration between a range of 0.0005–0.040 mg ml^−1^ (which is equivalent to 0.005–0.40% of unknown impurities and salicylic acid with respect to acetylsalicylic acid content). All the validation parameters for linearity are listed in Table 2.

**Table 2:** Linearity parameters (regression analysis of calibration curves, *n* = 3).

Parameter	Result
Linearity range [mg ml^−1^]	0.0005–0.04
Slope	35,833,336
y-intercept	7142
Correlation coefficient (r^2^)	0.9997

### Accuracy

The accuracy of an analytical method is defined as the closeness of the result obtained to the true value^14^. Accuracy results were presented in Table 3. Recovery studies showed, that the confidence interval of the average recovery value is within the range of 97.0–103.0%. %RSD of the %recovery was within a range of 0.9 to 5.4%. The accuracy analysis also revealed that the parameter values of regression line of relationship of theoretical and determined value were close to 1 for the slope coefficient and 0 for the Y-intercept.

**Table 3:** Accuracy.

Dosage ASA/GLY [mg mg^−1^]	Theoretical concentration [mg ml^−1^]	Found concentration [mg ml^−1^] (mean^a^ ± SD)	% Recovery (mean^a^ ± SD)	% RSD	Average recovery [%] (mean ± confidence interval)
75/40	0.0005 (0.005%)	0.00051 ± 0.00002	101.2 ± 4.6	4.5	100.4 ± 1.7
	0.005 (0.05%)	0.0050 ± 0.00019	99.5 ± 3.8	3.9	
	0.030 (0.30%)	0.0301 ± 0.00026	100.2 ± 0.9	0.9	
	0.040 (0.40%)	0.0402 ± 0.00041	100.5 ± 1.0	1.0	
100/40	0.0005 (0.005%)	0.00051 ± 0.00001	102.4 ± 2.9	2.8	101.2 ± 1.5
	0.005 (0.05%)	0.0050 ± 0.00005	100.8 ± 0.9	0.9	
	0.030 (0.30%)	0.0305 ± 0.00074	101.7 ± 2.5	2.4	
	0.040 (0.40%)	0.0400 ± 0.00131	100.0 ± 3.3	3.3	
150/40	0.0005 (0.005%)	0.00050 ± 0.00003	99.3 ± 5.4	5.4	99.3 ± 2.0
	0.005 (0.05%)	0.0051 ± 0.00006	101.0 ± 1.1	1.1	
	0.030 (0.30%)	0.0293 ± 0.00076	97.6 ± 2.5	2.6	
	0.040 (0.40%)	0.0398 ± 0.00113	99.4 ± 2.8	2.8	

^a^Mean value of three independent determinations.

### Precision

According to Naz et al.^15^ the precision of a method refers to the closeness of agreement (degree of scatter) between a series of measurements of a homogeneous sample. Precision can be considered as repeatability (intra-day precision) and intermediate precision (inter-day precision)^15^. Due to the confirmed specificity and accuracy of the method (in the presence of all 3 doses of placebo) precision determination was performed using model solutions corresponding to sample solution of dosage 150 mg ASA and 40 mg GLY (active substances and excipients).

Analysing the results of the precision, it was found that for all three concentration levels the value of intra-day and inter-day variance did not exceed assumed maximum value of 3.7%. The highest values of the inter-day variance (3.0%) and the intra-day variance (2.7%) were recorded for salicylic acid solution with a concentration of 0.0005 mg ml^−1^. %RSD in individual groups did not exceed 3.0% (Table 4).

**Table 4:** Precision values.

Theoretical concentration [mg ml^−1^]	Intra-day found concentration 1st measurement [mg ml^−1^] (mean^a^ ± SD)	Intra-day found concentration 2nd measurement [mg ml^−1^] (mean^a^ ± SD)	Intra-day variance^b^ (*n* = *6*)	Inter-day found concentration [mg ml^−1^] (mean^a^ ± SD)	Inter-day variance^b^ (*n* = 3 × 3)
0.0005 (0.005%)	0.00053 ± 0.00001 % RSD = 2.6	0.00051 ± 0.00001 % RSD = 2.8	2.7	0.00050 ± 0.00001 % RSD = 2.0	3.0
0.005 (0.05%)	0.0050 ± 0.0001 % RSD = 2.6	0.0050 ± 0.0001 % RSD = 1.5	2.1	0.0051 ± 0.0001 % RSD = 1.6	1.9
0.030 (0.30%)	0.0306 ± 0.0004 % RSD = 1.4	0.0305 ± 0.0003 % RSD = 0.9	1.1	0.0298 ± 0.0002 % RSD = 0.5	1.5

^a^Mean value of three independent determinations.

^b^Each value shows %RSD.

### Specificity

**Figure 2:**
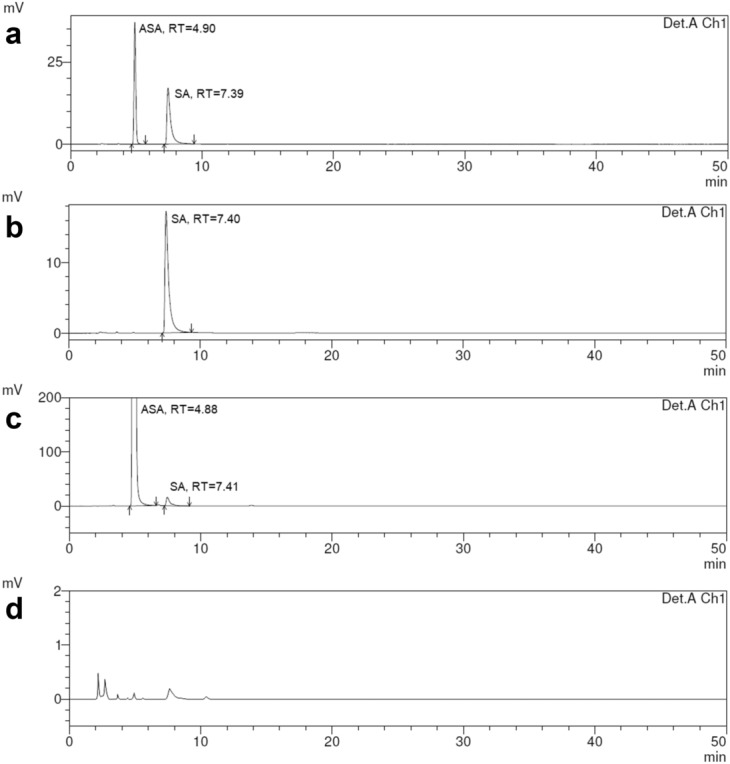
Exemplary chromatograms: (**a**) SSS solution, (**b**) reference solution, (**c**) solution containing reconstituted tablet (75 mg ASA, 40 mg GLY) spiked with salicylic acid at a concentration of 0.05% d) placebo of dosage 100 mg ASA and 40 mg GLY solution.

Obtained chromatograms confirmed method specificity. Any interfering peaks derived from the placebo, glycine, mobile phase, sample solvent was observed. Exemplary chromatograms are presented in Fig. 2.

### Robustness

According to authors^14^ robustness of an analytical method can be explained as an ability to remain unaffected by small changes in a method parameters. The robustness studies were performed to evaluate the effect of slight alternations of the stationary phase temperature on the chromatographic separation (Table 5). Performed variance analysis (α = 0.05) suggested that obtained mean values (n = 3) for each concentration level are not significantly different (F < F_cr_). The method has shown to be robust to slight increase of the stationary phase temperature. Moreover, robustness of the presented method was confirmed by satisfying results of intermediate precision.

**Table 5:** Robustness.

Theoretical concentration [mg ml^−1^]	Column temp. 25 °C found concentration [mg ml^−1^] (mean^a^ ± SD)	Column temp. 30 °C found concentration [mg ml^−1^] (mean^a^ ± SD)
0.0005 (0.005%)	0.00050 ± 0.00001 % RSD = 2.0	0.00053 ± 0.00002 % RSD = 3.5
0.005 (0.05%)	0.0051 ± 0.0001 % RSD = 1.6	0.0052 ± 0.0002 % RSD = 3.4
0.030 (0.30%)	0.0298 ± 0.0002 % RSD = 0.5	0.0301 ± 0.0001 % RSD = 0.5

^a^Mean value of three independent determinations.

### Range

Results obtained testify that validated method in a tested range of 0.005–0.40% of unknown impurities and salicylic acid with respect to acetylsalicylic acid content is linear, precise, accurate and robust to small increase of the stationary phase temperature.

### Limit of quantitation

Limit of quantitation (LOQ) testifies about method selectivity and can be defined as the minimum quantifiable concentration of an analyte^16^. On a basis of obtained results, LOQ was established as a concentration of 0.0005 mg ml^−1^. %RSD of six consecutive injections of salicylic acid standard solution at the concentration of 0.0005 mg ml^−1^ was found to be 2.5%, which fulfilled assumed acceptance criteria (%RSD not more than 10%).

### Standard and sample solutions stability

Stability of salicylic acid standard solution (reference solution) was confirmed for a solution stored in autosampler at a temperature of 10 °C for 18 h. Mean peak areas of 6 consecutive injections at the start of analysis and after 18 h did not differ significantly (test t and test F met). Stability of sample solution of tablets spiked with salicylic acid at a concentration of 0.30% was confirmed for a solution stored in autosampler at a temperature of 10 °C for 11 h. Mean peak areas of 3 consecutive injections at the start of analysis and after 11 h did not differ significantly (test t and test F met). Results of standard and sample solutions stability was presented in Table 6.

**Table 6:** Standard and sample solution stability.

Sample		Standard	
Initial mean peak area [mAU] (mean^a^ ± SD)	Mean peak area after predetermined time [mAU] (mean^a^ ± SD)	Initial mean peak area [mV s] (mean^b^ ± SD)	Mean peak area after predetermined time [mV s] (mean^b^ ± SD)
763.3 ± 2.1 % RSD = 0.3	766.0 ± 2.2 % RSD = 0.3	343,789 ± 408 % RSD = 0.1	344,073 ± 684 % RSD = 0.2

^a^Mean peak area of three injections.

^b^Mean peak area of six injections.

### Forced degradation

Forced degradation studies usually are performed to establish degradation pathways of drug substances as well as drug products. Furthermore, an important factor of this study is to differentiate degradation products, which are generated from placebo and which are related to drug substance^17^. The summary of degradation studies was presented in Table 7.

**Table 7:** Summary of degradation studies of ASA and excipients.

Degradation conditions	Retention time of degradation products for ASA (min)	Retention time of degradation products for excipients (min)
Acidic degradation (25 ml of 0.5 M HCl, 1 h, 100 °C)	7.4 (SA), 13.0, 19.4	–
Alkaline degradation (25 ml of 0.5 M NaOH, 1 h, 100 °C)	7.4 (SA), 33.9	3.8, 4.2, 6.9
Neutral degradation (25 ml of H_2_O, 1 h, 100 °C)	7.4 (SA), 13.0, 19.0, 34.5	–
Oxidative degradation (25 ml of 3% H_2_O_2_, 1 h, 100 °C)	7.4 (SA), 13.0	–
Photolytic degradation (UV, 8 h)	7.4 (SA), 6.5, 11.4, 12.9	–
Dry heat degradation (105 °C, 2 h)	7.4 (SA), 6.5, 11.4, 12.9, 34.2, 36.1	–

As a result of a reaction of aqueous solution of hydrochloric acid with acetylsalicylic acid, salicylic acid and single unknown impurity with retention times of 13.0 and 19.4 were recorded. On the other hand, the analysis of placebo of all doses showed that placebo substances did not decompose under the influence of 0.5 M HCl.

In the case of interaction of 0.5 M NaOH with acetylsalicylic acid, salicylic acid and a single unknown impurity with a retention time of 33.9 were recorded. Placebo of doses 75 mg ASA and 40 mg GLY as well as 150 mg ASA and 40 mg GLY, containing microcrystalline cellulose and maize starch, were decomposed under the influence of 0.5 M NaOH. All peaks derived from degradation products had retention times lower than salicylic acid (7.4 min). Placebo of dosage 100 mg ASA and 40 mg GLY, containing potato starch and talc did not decompose under the influence of 0.5 M NaOH.

Acetylsalicylic acid under the influence of boiling water has significantly decomposed into salicylic acid. Also, the appearance of individual peaks of unknown impurities was observed with retention times 13.0, 19.0 and 34.5. However, the excipients did not decompose in the aquatic environment into products appearing in the chromatograms.

Acetylsalicylic acid treated with 3% hydrogen peroxide significantly degraded into salicylic acid and a single unidentified impurity with retention time of 13.0 min. For excipients, no changes were observed under the influence of the analysed stressing factor.

Slight hydrolysis of acetylsalicylic acid under the influence of UV to salicylic acid occurred. Single unknown impurities were also observed with the retention times of 6.5, 11.4 and 12.9. The excipients did not undergo degradation under the influence of UV.

Acetylsalicylic acid was slightly decomposed to salicylic acid and unknown impurities with retention times of 6.5, 11.4, 12.9, 34.2 and 36.1, due to the increased temperature. Excipients did not degrade when exposed to increased temperature.

The forced degradation study confirmed that there was no merging of the peaks of the active ingredients nor salicylic acid with those of any other degradation products.

The obtained results of ASA degradation were in agreement with the results described by Yenduri and Navuluri^18^, who analysed forced degradation products of ASA by newly developed HPLC method. The authors stated that ASA was affected by acidic, alkaline, oxidative, thermal as well as photo degradation conditions.

## Conclusions

A method, adapted from European Pharmacopoeia, was validated successfully for the analysis of acetylsalicylic acid derived impurities in new tablet formulations with acetylsalicylic acid and glycine. It was confirmed that the method in a tested range of 0.005–0.40% of salicylic acid (equivalent to unknown impurities) with respect to acetylsalicylic acid content is linear, precise and accurate. The validated method is also robust to small changes of parameters, thus ensures repeatability of results in routine use.

The forced degradation study confirmed that there was no merging of the peaks of the active ingredients nor salicylic acid with those of any other degradation products. Moreover, any interfering peaks derived from the placebo, glycine, mobile phase, sample solvent were observed. The above results clearly confirm the suitable selectivity and sensitivity of the procedure. The proposed method, can be used in routine analyses of drug formulation with acetylsalicylic acid and glycine.
